# Small and Medium Enterprises and Global Risks: Evidence from Manufacturing SMEs in Turkey

**DOI:** 10.1007/s13753-020-00247-0

**Published:** 2020-02-12

**Authors:** Ali Asgary, Ali Ihsan Ozdemir, Hale Özyürek

**Affiliations:** 1 Disaster and Emergency Management, School of Administrative Studies, York University, Toronto, ON M3J 1P3 Canada; 2 Business Administration Department, Business School, Ankara Yıldırım Beyazıt University, Esenboğa Campus, Ankara, Turkey; 3 KOSGEB (Small and Medium Industry Development Organization), Ankara, Turkey

**Keywords:** Global risks, Risk assessment, Risk matrix, Small and medium enterprises, Turkey

## Abstract

This study investigated how small and medium enterprises (SMEs) in a country perceive major global risks. The aim was to explore how country attributes and circumstances affect SME assessments of the likelihood, impacts, and rankings of global risks, and to find out if SME risk assessment and rankings differ from the global rankings. Data were gathered using an online survey of manufacturing SMEs in Turkey. The results show that global economic risks and geopolitical risks are of major concern for SMEs, and environmental risks are at the bottom of their ranking. Among the economic risks, fiscal crises in key economies and high structural unemployment or underemployment were found to be the highest risks for the SMEs. Failure of regional or global governance, failure of national governance, and interstate conflict with regional consequences were found to be among the top geopolitical risks for the SMEs. The SMEs considered the risk of large-scale cyber-attacks and massive incident of data fraud/theft to be relatively higher than other global technological risks. Profound social instability and failure of urban planning were among the top societal risks for the SMEs. Although the global environmental and disaster risks were ranked lowest on the list, man-made environmental damage and disasters and major natural hazard-induced disasters were ranked the highest among this group of risks. Overall, the results show that SMEs at a country level, for example Turkey, perceive global risks differently than the major global players.

## Introduction

Small and medium enterprises (SMEs) face many small and large internal and external risks. While they can better control much of the internal risks through risk management and treatment measures, they are more vulnerable to external risks because these risks are often beyond their control, influence, radar, and capacity to manage. The World Economic Forum (WEF) has created, assessed, and monitored 30 global risks since 2005, using a survey of about 1000 major global stakeholders and players. By the WEF definition, a global risk is “an uncertain event or condition that, if it occurs, can cause significant negative impact for several countries or industries within the next 10 years” (World Economic Forum 2019, p. 100).

Small and medium enterprises are playing a vital role in local, national, and global economies and are very important in job and income generation (Chowdhury 2011; OECD 2014; Chatterjee et al. 2015). At least 90% of the firms in both developed and developing countries are SMEs (Mbuyisa and Leonard 2017). They account for 40–60% of GDP in developed and developing countries (Igwe et al. 2018) and generate about 40% of the global industrial production and 35% of the world’s exports (Sharma and Bhagwat 2006; Mbuyisa and Leonard 2017). Small and medium enterprises are the backbone of the European economy, with more than 99.8% of all non-financial businesses, 58% of total value added, and 66.8% of total employment (Briozzo and Cardone-Riportella 2012; European Commission 2015). In Japan, more than 99.7% of all firms are SMEs, they employ more than 70% of the workforce, and create more than 50% of all added value of the manufacturing industry (Yoshino and Taghizadeh-Hesary 2018). Small and medium enterprises comprised 99.8% of the firms in Turkey in 2014 and were involved in 55.1% of export and 37.7% of import (Kaya and Uzay 2017). Considering their size and roles in the national and global economies and the fact that the enhancement of the private sector’s resilience depends on risk reduction by SMEs (Chatterjee et al. 2015), more studies are needed to better understand various aspects of SME risk management.

Small and medium enterprises, like large corporations, face a significant number of risks, and their survival and resilience are important for national and global economies. However, SMEs are less prepared to manage the risks, and the institutional supports for them are rather weak (Han and Nigg 2011). Small and medium enterprises around the world, particularly in developing and emerging economies do not have strong risk management, business continuity, and crisis management cultures and systems in place (Asgary et al. 2013; Yuwen et al. 2016; Kaya and Uzay 2017). Most of SMEs not have the resources and expertise to focus on these activities and therefore are more vulnerable to internal and external risks and disruptive shocks (Leopoulos et al. 2006; Marks and Thomalla 2017). To minimize the impacts, it is important that SMEs become more aware of global risks, as well as assess, monitor, and enhance their risk management and business continuity management capacities (Güneş and Teker 2010; Brustbauer 2016; Kaya and Uzay 2017).

The goals of this study were twofold: (1) to examine whether country attributes and circumstances affect SME assessments of the likelihood, impacts, and rankings of global risks; and (2) to find out if SME risk assessment and rankings differ from global rankings. Small and medium enterprises in manufacturing in an emerging economy with global footprints were selected because, unlike the WEF that takes its samples from large international players, the sample SMEs are small individual players in the global economy and it is important to see how they view the global risks.

## Small and Medium Enterprises and Global Risks

The 2019 Global Risk Report by the World Economic Forum (WEF 2019) examines 30 important global risks that are classified into five categories: economic, environmental, geopolitical, societal, and technological (Table 1). These risks are evaluated annually based on 1000 global players and stakeholder views of the risks.

**Table 1:** The thirty global risks identified by the World Economic Forum. *Source*: World Economic Forum (WEF 2019)

Risk categories	Risks
Economic	Asset bubbles in a major economy
	Deflation in a major economy
	Failure of a major financial mechanism
	Failure/shortfall of critical infrastructure
	Fiscal crises in key economies
	High structural unemployment or underemployment
	Illicit trade
	Severe energy price shock
	Unmanageable inflation
Environmental	Extreme weather events
	Failure of climate-change mitigation and adaptation
	Major biodiversity loss and ecosystem collapse
	Major natural disasters
	Man-made environmental damage and disasters
Geopolitical	Failure of national governance
	Interstate conflict with regional consequences
	Failure of regional or global governance
	Large-scale terrorist attacks
	State collapse or crisis
	Weapons of mass destruction
Societal	Failure of urban planning
	Food crises
	Large-scale involuntary migration
	Profound social instability
	Rapid and massive spread of infectious diseases
	Water crises
Technological	Adverse consequences of technological advances
	Breakdown of critical information infrastructure and networks
	Large-scale cyber-attacks
	Massive incident of data fraud/theft

According to the 2019 WEF global risk report, extreme weather events, failure of climate-change mitigation and adaptation, natural disasters, data fraud or theft, cyber-attacks, man-made environmental damages and disasters, large-scale involuntary migration, biodiversity loss and ecosystem collapse, water crises, and asset bubbles in a major economy were ranked the top 10 global risks in terms of likelihood. Weapons of mass destruction, failure of climate-change mitigation and adaptation, extreme weather events, water crises, natural disasters, biodiversity loss and ecosystem collapse, cyber-attacks, critical information infrastructure breakdown, man-made environmental damages and disasters, and spread of infectious diseases were the top 10 global risks in terms of impacts. In both cases, three out of five environmental risks are among the top five risks and all five of them are in the top 10 risks (WEF 2019).

### Small and Medium Enterprises and Global Economic Risks

Global economic risks have significant implications for SMEs, particularly those in the manufacturing sector. Asset bubbles in a major economy can increase the production costs through inflation, wage increases and labor shortages, and access to financial resources that will impact the global economy (Zheng et al. 2010). Global financial crises cause substantial downturn in the formation of new SMEs, their performance, and their existence in the market. The 1997–1998 world financial and economic crisis severely impacted SMEs. As interest rates started to rise, many SMEs were bankrupted due to the credit crunch, tight monetary policies, and decline in domestic and international demands (Filardo 2011; Wehinger 2014). The number of bankrupted SMEs in South Korea, for example, particularly in the manufacturing sector, increased by nearly 100% from 1996 to 1998 (Gregory et al. 2002).

The 2008 economic crisis induced severe socioeconomic impacts worldwide and impacted SMEs in almost every economy, far beyond expectations, through fast domino effects that caused massive SME closures, downsizing, and reduced the number of new ventures (Chowdhury 2011; Sannajust 2014). Small and medium enterprises were under extreme pressures and experienced devastating decrease in demand and revenues, increased lay-offs, and stressful working environments (Kossyva et al. 2014). Close to 50% of the SMEs in Belgium and the Netherlands, for example, experienced extended delays in their receivables (Kossyva et al. 2014). Small and medium enterprises in the United States lost 2.8 million jobs (Gagliardi et al. 2013). During this global turmoil, Turkish SMEs were also impacted heavily (Karadag 2016).

During an economic crisis, SMEs are more vulnerable because of weak cash flow and financial structures, low equity reserves, limited adaptation potential and flexibility for downsizing, liquidation problems, too much dependency on external financial resources, tightened credit lines, payment delays on receivables, lack of resources, and lack of necessary skills to adopt or make necessary strategic decisions (Ates et al. 2013; Sannajust 2014; Wehinger 2014; Karadag 2016).

Failure of aging and insecure energy, transportation, and communications infrastructure can have major short- and long-term risks for SME performance and competitiveness. High structural unemployment lowers demand for goods and services and impacts SMEs significantly (Alegre and Chiva 2013). Illicit trade reduces SME competitiveness in the global market. In countries with higher levels of economic risk, SMEs have less of a chance to flourish (Mekinc et al. 2013). Energy is an important input for SME production and logistics. If energy prices are not manageable or controlable, SMEs face major uncertainties about energy costs and availability (Mulhall and Bryson 2014). Energy price shocks raise SME production costs (Kilian 2008) and compromise their individual and collective competitiveness in the global economy. It is mainly because SMEs are usually less flexible with respect to their energy sources and SMEs in the manufacturing sector are very energy intensive, that unpredicted fluctuations in energy prices impact them extensively. Energy price shock events have become more frequent and a consistent feature of the energy markets in recent years (Mulhall and Bryson 2014). As the global demand for energy increases, more shock events in the energy prices are expected. Finally, unmanageable high inflation rates at national and global levels pose risks to SMEs through higher interest rates (Cefis and Marsili 2006; Gül et al. 2010).

### Small and Medium Enterprises and Global Environmental and Disaster Risks

Small and medium enterprises around the globe, particularly those that are part of the global supply chains, are exposed to various types of global environmental and disaster risks that can have devastating impacts on SMEs (Auzzir et al. 2018). These enterprises are highly vulnerable to and not well prepared for most of the global environmental and disaster risks (Crichton 2006; Schaefer et al. 2011). They are vulnerable to environmental disaster risks on four fronts: capital, labor, logistics, and markets (Ballesteros and Sonny 2015). Environmental and disaster risk events can damage and disrupt the supply chain networks in which many SMEs are embedded. They can also damage SME assets, premises, and inventories, disrupt their operations, increase their production costs, and reduce their revenues and long-term growth potentials (Snyder and Shen 2006; Linnenluecke and Griffiths 2010, 2012; Asgary et al. 2012). Small and medium enterprises have limited capabilities to recover from these events and bring their operations, revenue, and profit back to pre-event conditions (Asgary et al. 2013). Considering the links that exist between climate change and extreme events, it is expected that these events will increase in the future (IPCC 2013). Small and medium enterprises face significant climate change-related environmental and regulatory risks (Schaefer et al. 2011). Major costly floods, severe heat and cold waves, heavy rains and extreme storms with higher frequency and intensity are observed globally. Extreme events not only cause disruptions and destruction to SMEs, but also create major challenges for their continuity of operations and future planning (Gunawansa and Kua 2014; Gasbarro et al. 2018). Studies show that few SMEs are adequately prepared for disaster risks. Small and medium enterprises are among the top underinsured sectors, and they usually do not conduct risk assessments or have business continuity plans (Wedawatta et al. 2010; Ye and Abe 2012). The 2011 floods in Thailand, for example, had major impacts on SMEs, and approximately 550,000 SMEs experienced direct and indirect damages, estimated at 71.1 billion Thai Baht per month, with 2.32 million jobs lost (Ye and Abe 2012). The 2011 Great East Japan Earthquake and the Thailand floods demonstrated that natural hazard-induced disasters can seriously inhibit the development of SMEs (Ye and Abe 2012; Auzzir et al. 2018).

Studies show that overall about 25% of SMEs do not reopen following a major disaster (Ballesteros and Sonny 2015). Of the US companies that experience disasters, for example, 43% never reopen, and another 29% close within 2 years (Weinhofer and Busch 2013; Ballesteros and Sonny 2015). Small and medium enterprises are worse off after disaster events compared to before disaster because they are relatively resource constrained, less resilient, are mainly informal and some of them do not fully comply or are not requested to follow standards and codes, lack necessary insurance, do not carry out risk assessments, and are often without business continuity plans (Ye and Abe 2012; UNDP 2013; Ballesteros and Sonny 2015; Halkos et al. 2018).

Being prone to multiple natural hazards such as flooding, earthquakes, and drought, natural hazards and disasters have affected SMEs in Turkey as well. The 1999 earthquake had significant economic impacts on the enterprise sector, ranging from USD 1.1 to 4.5 billion in damages (OECD 2000), most of it from the loss in manufacturing (USD 600 to 700 million). About 63.2% of the total manufacturing industry were damaged in five provinces, and 31,000 SMEs suffered heavy physical damages. Ezgi (2014) reported that the vast majority of SMEs had little preparedness before the earthquake and only 30% of them invested in insurance before the earthquake.

### Small and Medium Enterprises and Global Geopolitical Risks

A world of geopolitical instability and uncertainty is a major concern for all sectors and businesses, but more so for SMEs. Many of these risks are cross border with global consequences. While existing international political and economic agreements such as those of the World Trade Organization (WTO) are weakened by unilateralism, there is little evidence that new and better multilateralism agreements are replacing them (Pascual-Ramsay 2015; Asgary and Ozdemir 2019). Rather these agreements are being replaced by fragmentation, bilateralism, regionalism, as well as local and short-term interests (Pascual-Ramsay 2015; Asgary and Ozdemir 2019). The international economy and its key players, including SMEs, are becoming more exposed and vulnerable to existing and emerging geopolitical risks and uncertainties (Pascual-Ramsay 2015).

Studies show that terrorist attacks, for example, even though they are very small in terms of direct physical impact zones, have economic impacts that are often substantial and very extensive. Repeated terrorist attacks in one country not only impact the economy of that country but create spillover impacts for neighboring countries and the global economy. Terrorist attacks discourage foreign investments and capital inflows and cause significant loss of economic activities and international trade (Abadie and Gardeazabal 2008; Araz-Takay et al. 2009). These risks can also increase insurance, transaction, transportation, and security costs for SMEs.

Turkey as an emerging economy located in a geopolitically complex region (Middle East and North Africa), with several potentially failing neighboring states, and as a member of various types of regional agreements, has a unique situation in terms of geopolitical risks. Turkey has been suffering from terrorism and dealing with regional conflicts, both of which have had various impacts on the SMEs. The presence of terrorist activities has impacted the emergence and growth of SMEs and the overall economic performance in the country. Bilgel and Karahasan (2017) found that after the rise of terrorism, the per capita real GDP in Eastern and Southeastern Anatolia declined by about 6.6%. Other studies also found that terrorism has a major negative impact on foreign direct investments in Turkey (Omay et al. 2013).

### Small and Medium Enterprises and Global Societal Risks

Global societal risks have specific implications for SMEs. Failure of urban planning leads to declining cities, informal urban growth or sprawl, and poor and fragile infrastructure with significant social, environmental, and health issues (Asgary and Ozdemir 2019). Such urban environments are not able to adequately support enterpreneurship activities that can compete at national and global levels. Cities without efficient and interconnected transportation systems, with significant air pollution, and unaffordable land and housing prices are not attractive for entrepreneurship growth (TURSAB and TUADER 2017). But SME engagement in risk management and critical infrastructure protection is an effective way to reduce the impact of future disasters in urban areas (Chatterjee et al. 2015; Chatterjee et al. 2016). Food and water crises are other important global risks that can affect SMEs in several ways, particularly those in the agri-food business and those that are in water-intensive manufacturing sectors. Social instability as another global risk is not healthy for SME growth and competitiveness. Global pandemics such as the 2003 Severe Acute Respiratory Syndrome (SARS) pandemic and the 2009 H1N1 pandemic can have immediate direct and indirect impacts on SMEs. For example, SARS had major impacts on SMEs, particularly those in the tourism and hospitality sector in heavily impacted countries such as China, Canada, Thailand, and Hong Kong (Kuo et al. 2008). Studies have found that many SMEs do not recognize pandemics as a meaningful risk. Although governments have tried to raise awareness and provide resources to enhance pandemic preparedness by SMEs, awareness or concern and actual preparedness have not changed much, and most SMEs do not have appropriate preparedness and continuity plans for future pandemics (Watkins et al. 2008). Armed conflicts, interstate wars, natural hazards and disasters, and climate change are creating widespread involuntary and forced displacement around the globe. Population displacements have a range of economic, social, and political impacts on both source and host countries (Tumen 2016; Salgado-Gálvez 2018). The impacts of forced migration on SMEs have not been studied yet, but it may have both positive and negative impacts. At least SMEs can be considered a solution for some of these problems by providing job opportunities for displaced people. Turkey has received more than 4 million displaced people from Syria since the start of conflict in 2012 (Onur 2018).

### Small and Medium Enterprises and Global Technological Risks

Adverse consequences of technological advances could be very diverse and consequential for SMEs, especially those in the manufacturing sector. New technologies such as robotics, autonomous vehicles and drones, automation, smart phones, artificial intelligence, 3-D printing, cloud computing and big data, and new materials are among the new technologies that can have unintended consequences and risks for manufacturing SMEs. These technologies have the potential to reduce outsourcing. Studies predict that 47% of the jobs in the United States (much of them in SMEs) are at high risk of being automated over the next 20 years, especially in manufacturing, logistics, and administrative support (Pascual-Ramsay 2015). These advances will possibly reduce employment opportunities for workers in manufacturing SMEs and will challenge SMEs survival.

While information technology brings significant growth opportunities for SMEs through knowledge and information availability, business communication, cost savings and efficiency, improving decision making, responsiveness, and overall flexibility (Mbuyisa and Leonard 2017), technology also introduces risks, including data theft, disruptions, and cyber-attacks (Chacko and Harris 2006). Like other institutions, SMEs are dependent on internet and information technology and a substantial number of their sales and orders are handled through cyberspace and networks. Any major failure and disruption of the national and global information infrastructure and networks due to large-scale disaster events can have significant negative impacts on SMEs. Such disruptions can have severe consequences for SMEs that are very vulnerable and without adequate protection. Small and medium enterprises use these technologies in production and service delivery, distribution, sales, and marketing. Data breaches, cyber security, and intentional or accidental technological failures can disrupt or significantly damage the short- and long-term operation as well as the existence of SMEs.

## Methodology and Data

Following the WEF (2019), this study uses a qualitative risk assessment (QRA) approach. This will allow us to compare the results of the study with the global risk report results. Qualitative risk assessment is one of the most widely used risk assessment approaches because of its low cost and ease of use and it is quick to perform (Modarres 2006). In QRA, potential likelihoods and consequences are assessed using qualitative scales such as low, medium, and high. Qualitative risk assessment uses subjective likelihood and consequence values collected from experts and decision makers and, as such, they are not always perfect estimates and are subject to biases and heuristics (Talbot 2011). Assessed likelihoods and consequences for selected risks are then ploted in a two-dimensional space to generate a risk matrix. Various risk matrix forms and sizes have been reported in risk assessment reports. A risk matrix is used to visualize, compare, and rank different risks based on their locations in the matrix. Color coding is mostly used to show the importance of each risk. The risk matrix approach is also used for indicating possible risk control measures and to record the inherent, current, and target levels of risk (Hopkin 2012).

A risk matrix provides some basis for risk treatments and management. Risks that are located in the top right-hand corner of the risk matrix (often colored in red) have higher likelihoods and impacts. These risks are very critical and need to be controled. Risks that are in the lower (colored in green) and middle part (colored in orange or yellow) of the matrix should be monitored and checked regularly. Although the risk matrix method has been criticized by scholars and professionals (Cox 2008; Ni et al. 2010; Bao et al. 2017), it is an invaluable tool for fast, effective, and practical risk assessment (Talbot 2011).

Data were collected from a sample of manufacturing SMEs in Turkey. Small and medium enterprises in Turkey are categorized into three groups of micro, small, and medium-sized enterprises based on their employee numbers and annual revenues. Micro firms are those with less than 10 employees and less than USD 430,000 annual turnover. Small firms are those with less than 50 employees and less than USD 3.4 million annual turnover, and medium-sized firms are those with less than 250 employees and less than USD 17.2 million annual turnover (Karadag 2016).

To assess and evaluate the risks, a questionnaire survey, including 19 questions, was developed. Several questions collected general information about the production type, years in operation, city of operation, position of responder in the business, percent of production for export, percent of imported production materials, and export countries. In two sets of questions SME representatives provided their opinion about the consequences and likelihoods of global risks. Samples of a risk likelihood question and a risk consequences question are:

6. *Review the following global economic risks and give your opinion on the likelihood of these risks occurring in the manufacturing sector in Turkey over the next 10* *years.*
  *Critical infrastructure failure:*
    *very unlikely*
    *unlikely*
    *somewhat likely*
    *likely*
    *very likely*
7. *Please review the following global economic risks and give your opinion about the potential impacts/consequences of these risks on the manufacturing sector in Turkey over the next 10* *years.*
  *Critical infrastructure failure:*
    *minimal*
    *minor*
    *moderate*
    *severe*
    *catastrophic*

The questionnaire was designed and distributed using Google Form. Small and medium enterprises operating in the manufacturing sector (NACE Revision.02 in C Class through 10–33) were included in the population framework. These are SMEs in the NACE classes that are registered with the KOSGEB (Small and Medium Industry Development Organization, Turkey) and had an approved KOBİ (SME) certificate in 2017. The survey link was emailed to about 40,000 SMEs on 19 April 2019. Potential respondents were asked to complete the online survey by 3 May 2019. By the deadline, 217 completed responses had been received.

### Basic Characteristics of the Sample Small and Medium Enterprises

The sample covers SMEs in different manufacturing areas. After the unspecified “Other” manufacturing subgroup (39), SMEs in food products (22), textiles (22), machinery and equipment (22), furniture (20), fabricated metal (13), basic metal (9), wood products (9), rubber and plastic (9), electrical equipment (8), and chemical products (8) had the highest number of participants in this study. The questionnaire was completed by various individuals within each sample business, including managers (36), owners (20), accounting managers (13), financial managers (9), business partners (10), board members (3), engineers (6), and other employees (9). Sample SMEs are operating in 50 different cities and 7 geographic regions in Turkey, including Marmara (72), Central Anotolia (47), Aegean (29), Black Sea (24), Mediterranean (23), Eastern Anotolia (11), and Southeastern Anotolia (11).

The majority of the sample businesses (132) have been in operation for less than 10 years, only 34 have been in operation for 11 to 20 years, 29 between 21 and 30 years, and the rest (22) have been in business for more than 31 years. About 39.6% of the sample businesses were micro businesses, 37.8% small businesses, and about 22.6% were medium-sized enterprises. More than 60% of the SMEs export their products to varying degrees. They export to a large list of neighboring and European countries in particular. The sample SMEs also import some of their raw materials and equipment, and about 85% use imported products in their productions.

## Findings

Using the methodology on collected data respondents perceived likelihood of the global risks and their impacts were identified and risk values were calculated, and risk matrix was generated using the risk values. This section presents the key findings.

### Perceived Likelihood of Global Risks and their Impacts

**Fig. 1:**
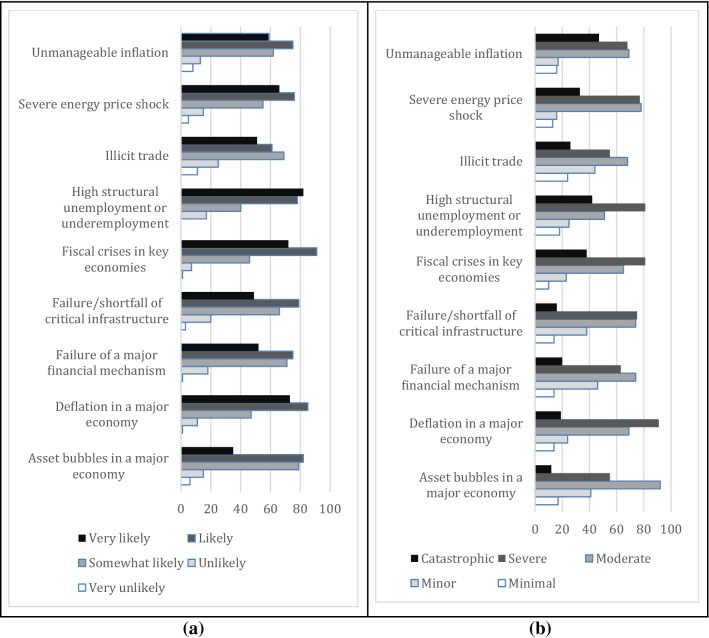
Stated likelihoods (**a**) and consequences (**b**) of the global economic risks by the surveyed small and medium enterprises in Turkey

Almost all global economic risks are perceived to have very high and high likelihoods by the sample Turkish SMEs (Fig. 1a). However, fiscal crises in key economies, high structural unemployment or underemployment, and severe energy price shock are among the most likely risks according to the sample enterprises. A majority of the SMEs thought that catastrophic and severe impacts can be expected from the economic risks, particularly unmanageable inflation, high structural unemployment or underemployment, and fiscal crises in key economies (Fig. 1b).

**Fig. 2:**
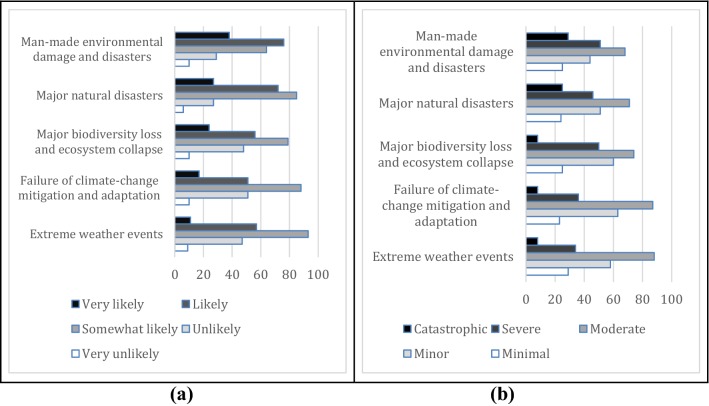
Stated likelihoods (**a**) and consequences (**b**) of the global environmental risks by the surveyed small and medium enterprises in Turkey

Global environmental risks seem to have relatively lower likelihoods to the sample SMEs, compared with the global economic risks (Fig. 2a). Man-made environmental damages caused by human and major natural hazards and disasters show higher perceived likelihood. The perceived impacts from these risks were scored lower as well. Among these risks, environmental damages caused by human are perceived to have slightly higher impacts for the sample businesses (Fig. 2b).

**Fig. 3:**
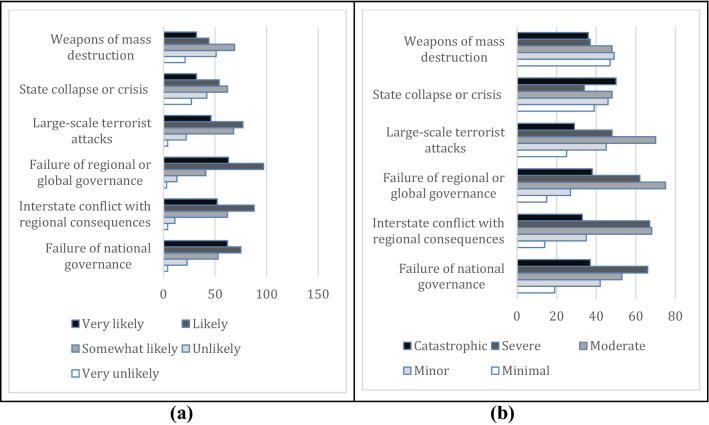
Stated likelihoods (**a**) and consequences (**b**) of the global geopolitical risks by the surveyed small and medium enterprises in Turkey

Figure 3a and b show the sample SME respondents’ opinion about the likelihoods and the consequences of the global geopolitical risks. Failure of national governance and failure of regional or global governance, and large-scale terrorist attacks have the highest average perceived likelihood in this risk category. However, the impacts are assessed to be higher for interstate conflicts with regional consequences, followed by the failure of national governance, and failure of regional or global governance.

**Fig. 4:**
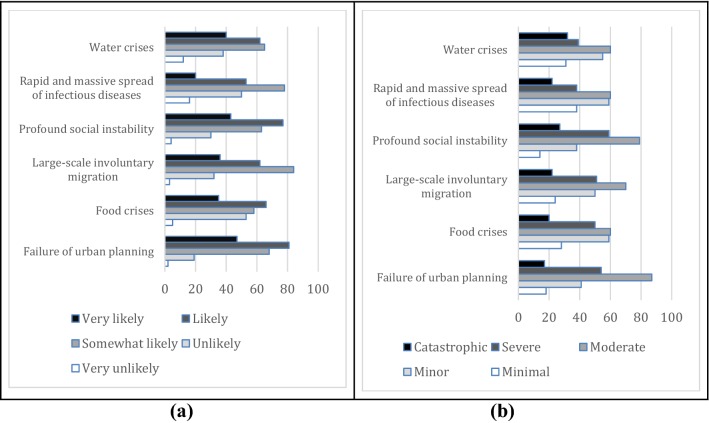
Stated likelihoods (**a**) and consequences (**b**) of the global societal risks by the surveyed small and medium enterprises in Turkey

Among global societal risks failure of urban planning and profound social instability were perceived to have the highest likelihood and impact averages among the sample businesses (Fig. 4a, b). While water crises seem to have a high perceived likelihood, sample businesses on average do not see it as having severe or catastrophic impacts on their operations.

**Fig. 5:**
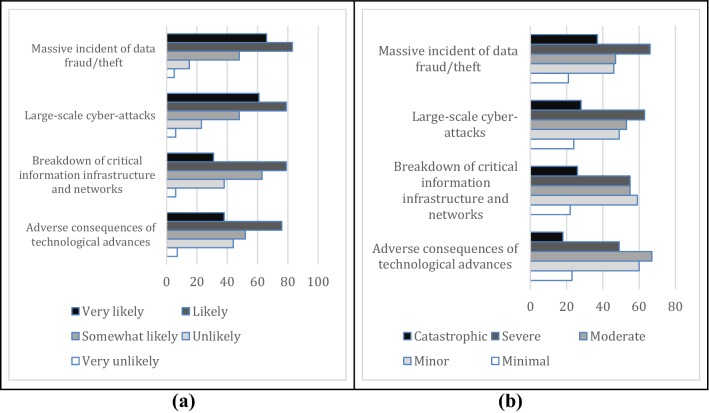
Stated likelihoods (**a**) and consequences (**b**) of the global technological risks by the surveyed small and medium enterprises in Turkey

Figure 5a and b present the stated likelihoods and consequences of the global technological risks. While the likelihood of all these risks is perceived to be high, large-scale cyber-attacks and large data fraud are among the top in this risk group. Although the means of the impacts are lower for most of these risks, except for the negative consequences of technological developments, more SMEs stated that the consequences of large-scale cyber-attacks and large-scale data fraud are expected to be severe and catastrophic.

### Risk Values

Risks can be calculated as the multiplication of likelihood by impacts (Table 2).

**Table 2:** Mean likelihood and impact of global risks as perceived by the surveyed small and medium enterprises in Turkey (N = 217)

Risk category	Risk	Likelihood	Impact	Risk = likelihood × impact	Rank
Economic	Asset bubbles in a major economy	3.58	3.02	10.8	16
	Deflation in a major economy	4	3.35	13.4	3
	Failure of a major financial mechanism	3.73	3.13	11.7	11
	Failure/shortfall of critical infrastructure	3.7	3.19	11.8	8
	Fiscal crises in key economies	4.04	3.53	14.3	1
	High structural unemployment or underemployment	4.04	3.48	14.1	2
	Illicit trade	3.53	3.07	10.8	17
	Severe energy price shock	3.84	3.47	13.3	4
	Unmanageable inflation	3.76	3.52	13.2	6
Environmental	Extreme weather events	3.06	2.7	8.3	30
	Failure of climate-change mitigation and adaptation	3.06	2.74	8.4	28
	Major biodiversity loss and ecosystem collapse	3.17	2.8	8.9	26
	Major natural disasters	3.4	2.99	10.2	21
	Man-made environmental damage and disasters	3.47	3.07	10.7	18
Geopolitical	Failure of national governance	3.77	3.28	12.4	10
	Interstate conflict with regional consequences	3.8	3.32	12.6	7
	Failure of regional or global governance	3.94	3.37	13.3	5
	Large-scale terrorist attacks	3.64	3.05	11.1	15
	State collapse or crisis	3.1	3.05	9.5	25
	Weapons of mass destruction	3.07	2.84	8.7	27
Societal	Failure of urban planning	3.7	3.05	11.3	14
	Food crises	3.34	2.88	9.6	24
	Large-scale involuntary migration	3.44	2.99	10.3	20
	Profound social instability	3.58	3.22	11.5	13
	Rapid and massive spread of infectious diseases	3.05	2.76	8.4	29
	Water crises	3.37	2.94	9.9	22
Technological	Adverse consequences of technological advances	3.43	2.9	9.9	23
	Breakdown of critical information infrastructure and networks	3.42	3.02	10.3	19
	Large-scale cyber-attacks	3.76	3.1	11.7	12
	Massive incident of data fraud/theft	3.88	3.24	12.6	9

**Fig. 6:**
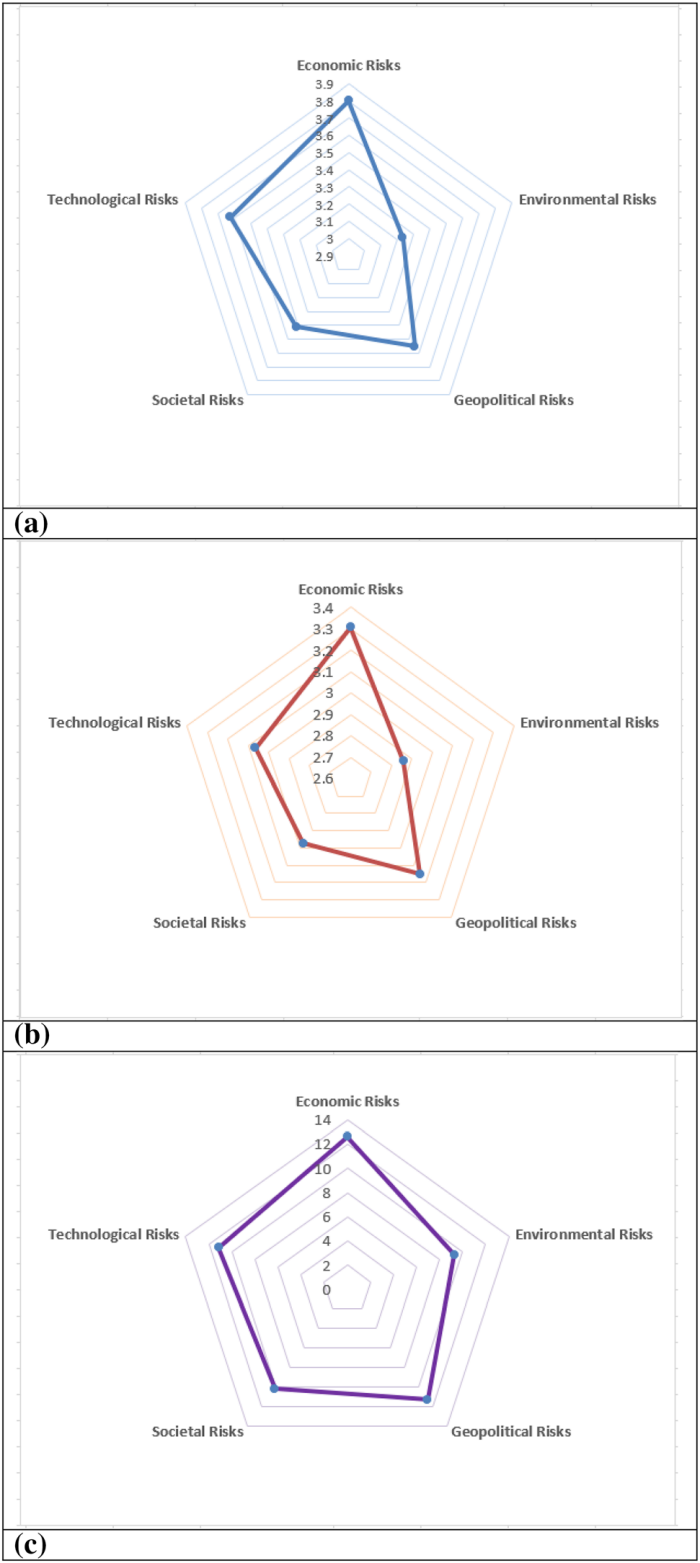
Radar diagram for global risk likelihoods (**a**), risk impacts (**b**), and risk values (likelihood × impact) (**c**) perceived by the surveyed small and medium enterprises in Turkey

Using the mean values of each risk category, economic and technological risks are perceived to have the highest likelihood levels followed by geopolitical risks (Fig. 6a). In terms of impacts, however, economic risks and geopolitical risks take the first and second ranks followed by technological risks. Societal and environmental risks are considered to have lower impacts (Fig. 6b). In terms of the overall risk, the results show that economic risks and geopolitical risks take the first and second place, followed by technological risks (Fig. 6c).

### Risk Matrix

Using the qualitative risk analysis methodology and perceived likelihood and impact data, a risk matrix was generated. Although the horizontal and vertical axes take the values 1 to 5, the matrix axes have been rescaled for better visualization. This risk matrix displays the means of stated likelihoods and consequences for each risk. Risks at the top right and in the red colored area are risks with higher than average likelihoods and consequences. Risks in the lower left part of the matrix and colored green are considered to be low.

**Fig. 7:**
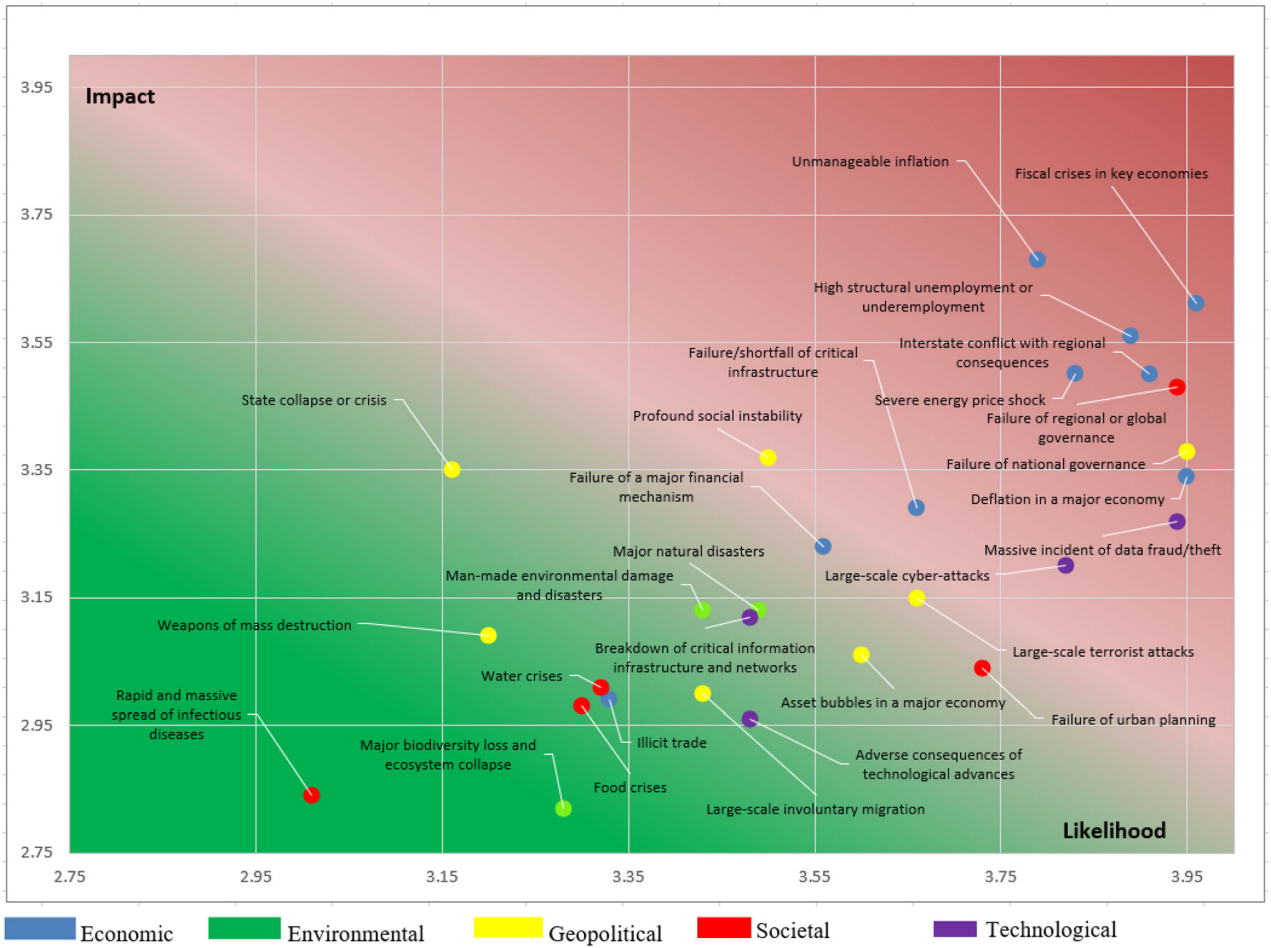
Risk matrix for the sample small and medium enterprises in Turkey

Figure 7 presents the resulting risk matrix for the 217 businesses and the 30 risks. Most economic risks are in the upper part of the risk matrix, followed by some geopolitical risks. Large-scale data fraud is the only technological risk that falls into the same area. Majority of environmental and societal risks, although scattered diagonally in the risk matrix, are in the lower part of the matrix.

## Discussion

This study examined the global risks from the perspective of manufacturing SMEs with global footprints in the emerging economy of Turkey. The main aim was to understand whether and to what extent country- and industry-specific contexts and conditions affect SMEs perceptions of the global risks. Key findings are discussed here.

First, overall the results suggest that regardless of the ranking, the global risks are of high concern for SMEs in Turkey. The average likelihood for all global risks is 3.56 and the average impact is 3.1. The minimum perceived likelihood (infectious desease) is 3.05 and the minimum perceived impact (severe weather events) is 2.7. These figures confirm that all global risks are of concern for the SMEs and have significant implications for them, particularly those in the manufacturing sector (Zheng et al. 2010).

Second, findings indicate that the SMEs’ perceived risks at the country level (Turkey) significantly varied from those perceived by the global companies in the global risk report (World Economic Forum 2019) (Table 3). While this study does not examine the underlying causes of these differences, it is evident that the SMEs’ major concerns are global economic and geopolitical risks, both in terms of the likelihoods and the impacts. Individual SMEs in Turkey have been exposed and impacted more by the global economic risks than other risks. Our findings are consistent with a few different but related research conducted by Gül et al. (2010), Topçu (2013), and Deloitte (2017) that economic and financial risks such as devaluation of the Turkish Lira, interest rate risk, breakdown in cash flow or liquidity risk, credit risk, and increase in input prices were the key risks that businesses are facing in Turkey.

**Table 3:** Top 10 global risks in terms of likelihoods and impacts for the sample small and medium enterprises in Turkey

Rank	Likelihood	Impact
1	Fiscal crises in key economies	Fiscal crises in key economies
2	High structural unemployment or underemployment	Unmanageable inflation
3	Deflation in a large economy	High structural unemployment or underemployment
4	Interstate conflict with regional consequences	Severe energy price shock
5	Massive incident of data fraud/theft	Interstate conflict with regional consequences
6	Severe energy price shock	Deflation in a large economy
7	Failure of regional or global governance	Failure of regional or global governance
8	Failure of national governance	Failure of national governance
9	Unmanageable inflation	Massive incident of data fraud/theft
10	Large-scale cyber-attacks	Profound social instability

Third, it is not surprising that SMEs’ highest perceived risks are economic and geopolitical risks. Studies demonstrate that financial and economic crises cause substantial downturn in the formation of new SMEs and the performance and survival of the existing ones in the market (Gregory et al. 2002; Zheng et al. 2010; Chowdhury 2011; Filardo 2011; Kossyva et al. 2014). Small and medium enterprises are very vulnerable to economic and financial crises as they are forced to close, downsize, and reduce the number of new ventures due to sharp decrease in demand and revenues (Ates et al. 2013; Sannajust 2014; Wehinger 2014). In today’s global economy, Turkish SMEs are not exempt from this, and they have been frequently impacted by such risks in the past two decades as well (Karadag 2016). Moreover, giving high likelihood and high impact values for financial crises and other economic risks can be explained by the fact that Turkish economy has deficit in international trade (Abbasoğlu et al. 2019) and highly rely on external energy sources such as oil and natural gas.

Fourth, failure of regional or global governance and failure of national governance are the geopolitical risks that are among the top perceived risks by the SMEs in this study. These risks have been largely felt by Turkish SMEs in recent years. Turkey has been in close proximity to a number of regional conflicts with potential impacts on the SMEs (Omay et al. 2013; Bilgel and Karahasan 2017) and because of their vulnerability (Pascual-Ramsay 2015) and awareness of these risks, such risks are perceived highly both in terms of the likelihoods and the impacts.

Fifth, the sample SMEs also consider the likelihood of large data fraud/theft and large-scale cyber-attacks to be high. This is possibly due to the increasing dependency of the SMEs to the internet and the increasing number of cyber attacks and data theft in recent years (Mbuyisa and Leonard 2017). While SMEs do not consider the impacts of these risks as high as their likelihoods, still these risks can cause disruptions and severe consequences to them, particularly because they are not well equipped to manage these risks. A recent report published by Allianz (2019) confirms that SMEs in Turkey increasingly recognize their cyber vulnerability and risks.

Finally, the relatively lower perceived likelihoods and impacts of the global risks by the sample SMEs can be attributed to the fact that small businesses may not be directly and highly impacted by distance environmental risks such as major natural hazard-induced disasters and that the awareness about some of the environmental risks among the SMEs may be lower than other risks. Moreover, Turkey has not experienced a major natural hazard-induced disaster in the past 20 years, and major weather events have been very local.

## Conclusion

The results of this study indicate the importance of addressing global risk assessments by SMEs. As more and more SMEs are connected with the national and global economies, their awareness about theses risks and the impacts that they could have for them will increase. This awareness can help SMEs to take these risks into consideration and prepare themselves for such risks. This study highlighted that SMEs’ perceptions of the global risks are different from the businesses that operate at large scale at the global level. It also demonstrated that country’s circumstances can affect SMEs’ assessments of the likelihood, impacts, and rankings of global risks. It demonstrated that SMEs are more concerned about economic risks and risks that directly impact economic systems and variables, particularly geopolitical risks. Environmental risks, while important, are not at the top of the list for SMEs. Considering the significant role that SMEs play in local and national economies and the fact that they are concerned most about global economic and geopolitical risks, it can be argued that efforts towards lowering global economic and geopolitical risks can significantly benefit SMEs.

Since Turkey’s SMEs have been in a relatively unique situation in the past two decades with respect to some of the major global risks, similar studies in countries in other parts of the world may shed more light on how country contexts and type and size of businesses impact SMEs’ perceptions of global risks. It was beyond the scope of this study to examine the SMEs’ risk and business continuity actions taken to manage and mitigate the risks. Future studies can also investigate whether and how SMEs prepare themselves for global risks.
